# A case of checkpoint inhibitor-induced celiac disease

**DOI:** 10.1186/s40425-019-0694-x

**Published:** 2019-08-05

**Authors:** Dana Alsaadi, Neil J. Shah, Aline Charabaty, Michael B. Atkins

**Affiliations:** 1 Department of Internal Medicine, MedStar Georgetown University Hospital, Washington, DC USA; 2 Lombardi Comprehensive Cancer Center, MedStar Georgetown University Hospital, 3800 Reservoir Road, NW, Washington, DC 20007 USA; 3 Sibley Memorial Hospital, Johns Hopkins University, 5255 Loughboro Road NW, Washington, DC 20016 USA

**Keywords:** Nivolumab, Ipilimumab, Immune checkpoint inhibitor, Celiac disease, Immune-related adverse event

## Abstract

**Background:**

Immune checkpoint inhibitors (ICIs) have now become standard of care treatment for many malignancies. ICIs are associated with unique immune mediated adverse events (irAEs) due to dysregulation of immune activation. As treatment with ICIs is becoming more common, rare irAEs are also being recognized. Here we report a case of ICI-induced celiac disease.

**Case:**

A 74-year-old Caucasian female with metastatic renal carcinoma received second line nivolumab (anti-PD1 antibody) after initial disease progression on sunitinib. Ipilimumab was added after she failed to respond to six cycles of nivolumab monotherapy. One week after her first cycle of combination treatment, she presented with nausea, vomiting, grade 1 diarrhea, and weight loss. She underwent endoscopy, which showed bile stasis in the stomach, normal appearing stomach mucosa, and nonbleeding erythematous mucosa in the duodenal bulb. Stomach biopsy showed moderate active chronic gastritis. Duodenal biopsy showed moderate chronic active duodenitis with focal neutrophilic cryptitis, mucosal erosions, villous atrophy, mildly increased intraepithelial lymphocytes, and moderate chronic inflammation in the lamina propria pathognomonic of celiac disease. Symptoms improved with gluten-free diet, twice-daily omeprazole and anti-emetics and she was able to continue on treatment.

**Conclusions:**

There has been only one published case reporting ICI-induced celiac disease. Our case report highlights a rare irAE (celiac disease) associated with ICI treatment. It is unclear whether the patient had previously undiagnosed celiac disease or whether ICIs triggered her enteritis. Our patient was able to continue treatment with ICIs with dietary modifications, suggesting correct diagnosis is critical for optimal patient outcome.

## Background

Immune checkpoint inhibitors have become a mainstay in the treatment of metastatic malignancies, such as melanoma and lung cancer, as they increase the survival of patients who failed conventional therapies. Nivolumab is a human monoclonal IgG4 antibody that inhibits the programmed death-1 (PD-1) pathway, which is an important regulator of the induction and maintenance of peripheral tolerance against malignant cells [1–3]. When a tumor cell ligand binds the PD-1 receptor, a co-inhibitory molecule expressed on T-cells, it down-regulates the cellular immune response. Nivolumab restores T-cell immunity by interfering with co-inhibitory molecule induced T cell tolerance to tumor cells. Ipilimumab is a human monoclonal IgG1k antibody that blocks cytotoxic T-lymphocyte–associated antigen 4 (CTLA-4). CTLA-4 is a T-cell co-inhibitory molecule that outcompetes the co-stimulatory molecule CD28 for binding to B7 on antigen-presenting cells, thereby down-modulating cytotoxic T-cell function and allowing cellular proliferation. Ipilimumab binds to CTLA-4, which is induced on activated T cells preventing down-regulation of cytotoxic T cell function. In addition, CTLA-4 is constitutively expressed on regulatory T-cells, where the binding of ipilimumab leads to antibody dependent cellular cytotoxicity (ADCC), thereby eliminating a major immunosuppressive factor in the tumor microenvironment [4].

While ICIs have revolutionized metastatic cancer treatment, they produce unique immune-related adverse events that include diarrhea and colitis. These side effects vary in time of onset, but typically occur after the first few doses of ICI. ICI enterocolitis can be most effectively managed when diagnosed early and immunosuppressive therapy is initiated within the first five days of symptoms [4]. Unrecognized or undertreated ICI-induced colitis can lead to bowel perforation and fatal outcome [5]. The choice of immunosuppressive therapy depends on the severity of irAE (grading is based on the common terminology criteria for adverse events (CTCAE) Version 5.0, 2017) [6]. For grade 1 diarrhea (which is an increase of less than 4 stools per day over the patient’s baseline), symptomatic treatment with loperamide, rehydration, electrolytes substitution is advised. For grade 2 diarrhea, steroid therapy with either budesonide or 1 mg/kg prednisone is recommended. In cases of severe diarrhea (grade 3 and above), high dose IV corticosteroids such as methylprednisolone or dexamethasone should be given. Grade 3 is defined as ≥7 stools per day over baseline and necessitating hospitalization for IV fluids. If no improvement is seen after 3–5 days of high dose steroids, a dose of infliximab (IFX), a tumor necrosis factor-α (TNF-α) inhibitor, or occasionally vedolizumab, an antibody to α4β7-integrin which facilitates T-cell trafficking into the gut mucosa, have been successfully used to achieve a clinical resolution of the ICI-induced colitis [7–10].

As treatment with ICIs is becoming more common, rare irAEs are also being recognized. While colitis is the main cause of diarrhea in ICI-treated patients, here we report a case of diarrhea due to ICI-induced celiac disease.

### Case report

A 74-year-old Caucasian female with metastatic renal carcinoma received second line nivolumab after initial disease progression on sunitinib. She experienced grade 1 AST/ALT elevation and continued treatment. Ipilimumab was added after she failed to respond to six cycles of nivolumab monotherapy. One week after her first cycle of combination treatment, she presented with nausea, vomiting, and weight loss. She also had grade 1 diarrhea, which was treated symptomatically with loperamide.

**Fig. 1:**
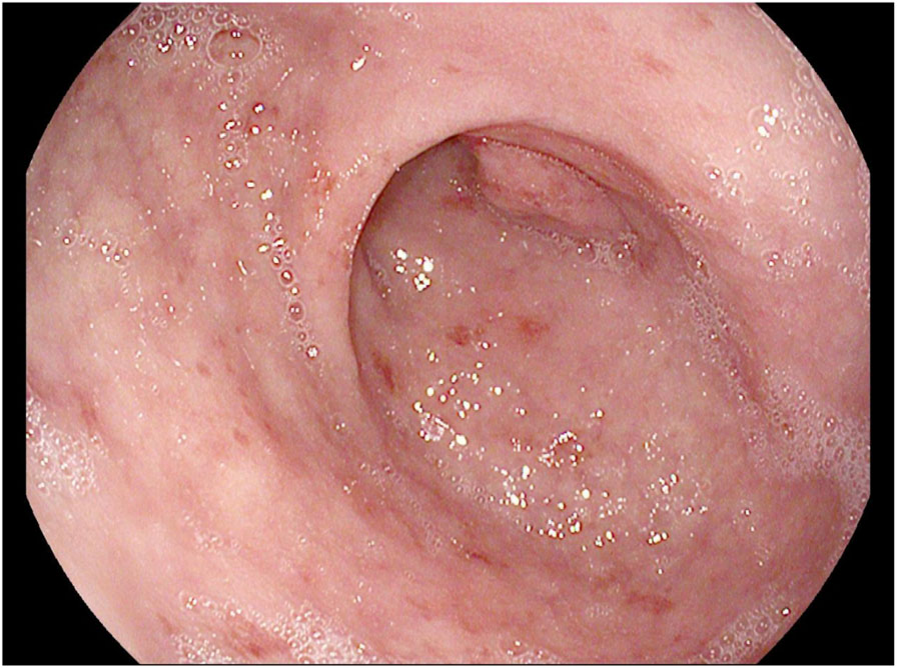
Endoscopic Picture of Duodenum. Inflammation in the duodenal bulb with non-bleeding erythematous mucosa

**Fig. 2:**
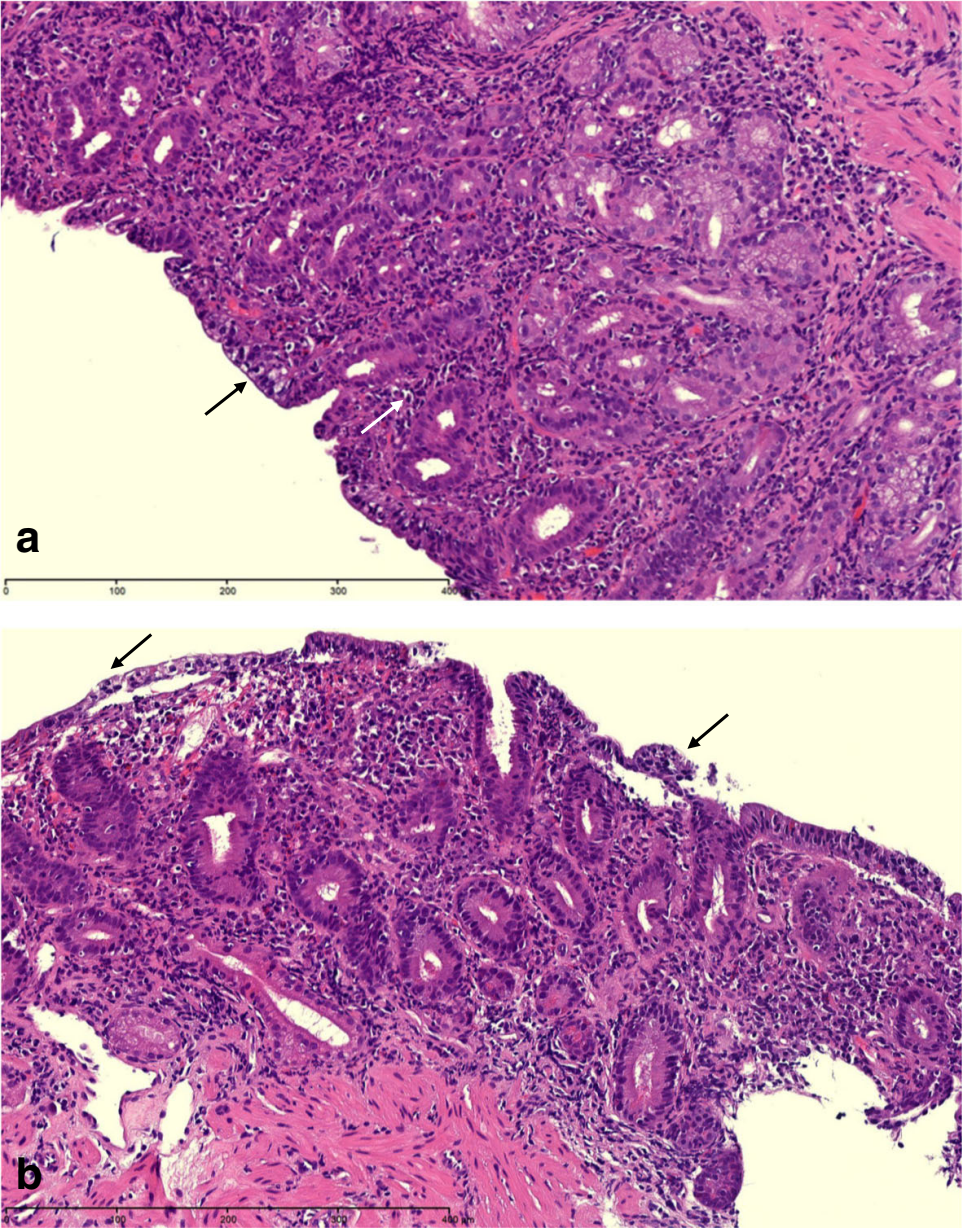
Duodenal biopsy. a. Villous atrophy (black arrow) and chronic inflammation in the lamina propria with diffuse intraepithelial lymphocytosis (white arrow). b. Mucosal erosions (black arrows)

She underwent upper endoscopy, which showed bile stasis in the stomach, normal appearing stomach mucosa, and nonbleeding erythematous mucosa in the duodenal bulb (Fig. 1). The second part of the duodenum showed normal mucosa without abnormalities. Stomach biopsy showed moderate active chronic gastritis. Giemsa stain for *Helicobacter Pylori* was negative. Duodenal bulb biopsy showed moderate chronic active duodenitis with focal neutrophilic cryptitis, mucosal erosions, villous atrophy, mildly increased intraepithelial lymphocytes, and moderate chronic inflammation in the lamina propria, suggestive of celiac disease (Fig. 2). Immunohistochemistry was performed with antibodies against CD3, CD8, and CD56 to rule out celiac disease because of villous atrophy. CD3 immunostains showed mildly increased intraepithelial T cells, between twenty and thirty lymphocytes per hundred epithelial nuclei in the villi, but not to the usual degree seen in celiac disease (which is defined as having greater than forty lymphocytes per hundred epithelial nuclei). Stains were negative for increased CD8-positive T cells and CD56-positive Natural Killer cells.

Serum tissue transglutaminase IgA antibody level was elevated to 12 unit/mL (normal 0–3), which was diagnostic for celiac disease. The patient was started on a gluten-free diet for celiac disease, omeprazole 40 mg by mouth twice daily for gastritis, and the anti-emetics ondansetron and metoclopramide as needed. Symptoms improved, and she was able to continue on treatment.

The patient experienced a recurrence of symptoms, however, that was worse after each ICI infusion. Eight weeks after her endoscopy, she was also started on budesonide 9 mg by mouth daily and prochlorperazine three times a day with meals. Symptoms improved with budesonide. The patient also exhibited ICI-induced hypothyroidism and pancreatitis, with an increase in lipase from baseline 77 to 400. She was treated with pancreatic enzymes and thyroid replacement.

Interval imaging was concerning for progression of disease, and the patient discontinued nivolumab and ipilimumab after receiving 4 cycles of combination therapy. She continued gluten-free diet and was able to gain weight. She was tapered off of budesonide over a period of 6 months.

## Discussion

Immune-checkpoints inhibitors have revolutionized the treatment of metastatic malignancies; however, they can trigger various organ-specific irAEs, such as nausea and diarrhea, which can limit their use even with evidence of regression of the underlying malignancy. One third of patients treated with ipilimumab, an anti-CTLA-4 antibody, develop diarrhea and 16% of patients will go on to develop severe colitis, which can lead to perforations (0.5%) and/or colectomy [4, 11]. Nivolumab, an anti-PD-1 antibody, causes diarrhea in 8–19% of patients, of whom only 1% experience grade 3 or 4 diarrhea [5, 12, 13]. Patients treated with a combination of ipilimumab and nivolumab have a 44% chance of developing diarrhea, with grade 3 diarrhea accounting for 20% of all cases [12]. Typically, the onset of diarrhea occurs 6 weeks after initiation of treatment, but can be delayed up to 6 months after the last dose of ICI [13]. Patients can also experience other irAEs separately or concomitantly, such as thyroiditis, myositis, and hepatitis, which suggests a systemic auto-immune like reaction to ICIs.

While colitis is the most common cause of diarrhea in the ICI-treated patient, alternative etiologies of diarrhea must also be considered. There has been only one published case reporting ICI-induced celiac disease due to ipilimumab [14]. Our case report highlights a rare irAE, celiac disease, associated with ICI treatment. It is unclear whether the patient had previously undiagnosed celiac disease or whether ICIs triggered her enteritis, but the patient was asymptomatic prior to initiation of ICI. Given that she also exhibited other well-characterized concomitant irAEs such as pancreatitis and hypothyroidism, we suspect that her celiac disease was triggered by ICIs. The initiation of ipilimumab in particular seemed to trigger her symptoms, which is in concordance with the literature that has shown the strong immunogenic effects of ipilimumab compared to other ICIs. The patient’s celiac diagnosis was coincidental; given that her diarrhea was of a lower grade, she was treated symptomatically with loperamide. Upper endoscopy was mainly performed for her nausea and vomiting.

The pathogenesis of celiac disease is due to gluten-mediated activation of intestinal CD4+ T cells in the lamina propria. Gliadin peptides from gluten are converted by tissue transglutaminase (TTG) to a form that increases their affinity for HLA-DQ2 and HLA-DQ8 molecules and results in enhanced antigen presentation. Antigen presenting cells activate CD4+ T-helper cells in the lamina propria, causing expansion of cells that produce antibodies to gliadin and TTG [15]. The histologic hallmarks of celiac disease on small bowel biopsies are intraepithelial lymphocytosis, lymphoplasmacytic inflammation of the lamina propria, villous atrophy, and crypt hyperplasia [16].

While the pathogenesis of immune-mediated colitis is not well understood, CTLA-4 blockade leads to T-cell activation that increases secretion of CD4 T-helper cell cytokines and cytolytic CD8 T-cell tissue infiltration [17]. In contrast to celiac disease, ICI-induced colitis usually presents with an array of histologic findings. Usually biopsies demonstrate features of acute active colitis such as intraepithelial neutrophilic infiltrates or crypt abscesses, increased mononuclear cells in the lamina propria [18]. Both celiac disease and ICI colitis show increased apoptotic cells in crypts. Interestingly a subset of ICI colitis patients may demonstrate intraepithelial or basal lymphocytes, excess plasma cells in the lamina propria, and lymphocytic cryptitis on colonic biopsy, which is more consistent with findings in chronic colitis [19].

As histologic features may overlap, clinical features are crucial for differentiation of distinct diseases. This patient tested positive for tissue transglutaminase antibodies; the serum ELISA anti-TTG test has 93% sensitivity and 98% specificity for a celiac diagnosis [15]. Typically celiac disease is diagnosed by biopsy when there are greater than forty lymphocytes per hundred epithelial nuclei in the villi. Thus, although the patient’s histology showed less than thirty lymphocytes per hundred epithelial nuclei, a diagnosis of early celiac disease can be made in conjunction with a positive serology. Gastroenterologists must consider histologic and endoscopic features, clinical symptoms, and laboratory findings such as celiac serology and genetic testing to arrive at the correct diagnosis.

Our patient was able to continue treatment with ICIs with dietary modifications, suggesting correct diagnosis is critical for optimal patient outcome. As highlighted by this case, active inflammation can affect the small bowel and/or upper GI tract alone. For a patient with symptomatic diarrhea, evaluation shouldn’t be limited to colonoscopy alone, and biopsies should be done to look for microscopic evidence of inflammation even if the mucosa of the GI appears normal.

Both early recognition and initiation of the appropriate treatment of irAEs are crucial to relieve symptoms, avoid complications, and when indicated enable continued ICI therapy. While enterocolitis is by far the most common cause of diarrhea, we here report a case of celiac disease induced by ICI therapy. In a patient with symptomatic diarrhea following initiation of ICIs, infectious pathology should be ruled out followed by initiation of systemic corticosteroids. In patients with unusual features and/or failure to respond to steroid treatment, consideration should be given to a full endoscopic work up including a colonoscopy and exam of the terminal ileum and an upper endoscopy with biopsies in order to discern the underlying etiology. This case of ICI-induced celiac disease demonstrates that multidisciplinary collaboration among oncologists, gastroenterologists, and pathologists is crucial for correct diagnosis and treatment.

## Data Availability

Not applicable.

## Abbreviations

CTCAE: Common Terminology Criteria for Adverse Events
ICI: Immune checkpoint inhibitor
irAE: immune related adverse event
TTG: Tissue transglutaminase

## References

[1] Sundar R, Cho B, Brahmer JR, Soo RA (2015). Nivolumab in NSCLC: latest evidence and clinical potential. Ther Adv Med Oncol.

[2] Motzer RJ, Rini BI, McDermott DF (2015). Nivolumab for metastatic renal cell carcinoma: results of a randomized phase II trial. JCO..

[3] McDermott DF, Atkins MB (2013). PD-1 as a potential target in cancer therapy. Cancer Med.

[4] Graziani G, Tentori L, Navarra P (2012). Ipilimumab: a novel immunostimulatory monoclonal antibody for the treatment of cancer. Pharmacol Res.

[5] Abdel-Rahman O, Helbling D, Schmidt J (2017). Treatment-related death in cancer patients treated with immune checkpoint inhibitors: a systematic review and meta-analysis. Clin Oncol.

[6] US Department of Health and Human Services. National Cancer Institute. Common terminology criteria for adverse events (CTCAE). 2017. https://ctep.cancer.gov/protocoldevelopment/electronic_applications/docs/CTCAE_v5_Quick_Reference_8.5x11.pdf. Accessed 24 Feb 2019.

[7] Hodi FS, O'Day SJ, McDermott DF, Weber RW, Sosman JA, Haanen JB (2010). Improved survival with ipilimumab in patients with metastatic melanoma. N Engl J Med.

[8] Robert C, Long GV, Brady B, Dutriaux C, Maio M, Mortier L (2015). Nivolumab in previously untreated melanoma without BRAF mutation. N Engl J Med.

[9] Rastogi P, Sultan M, Charabaty AJ, Atkins MB, Mattar MC (2015). Ipilimumab associated colitis: an IpiColitis case series at MedStar Georgetown University Hospital. World J Gastroenterol.

[10] Diana P, Mankongpaisarnrung C, Atkins MB, Zeck JC, Charabaty A (2018). Emerging role of Vedolizumab in managing refractory immune checkpoint inhibitor-induced enteritis. ACG Case Rep J.

[11] Gupta A, De Felice KM, Loftus EV, Khanna S (2015). Systematic review: colitis associated with anti-CTLA-4 therapy. Aliment Pharmacol Ther.

[12] Shoushtari AN, Friedman CF, Navid-Azarbaijani P (2017). Measuring toxic effects and time to treatment failure for Nivolumab plus Ipilimumab in melanoma. JAMA Oncol.

[13] Villadolid JAA (2015). Immune checkpoint inhibitors in clinical practice: update on management of immune-related toxicities. Translational Lung Cancer Research.

[14] Gentile NM, D’Souza A, Fujii LL, Wu TT, Murray JA (2013). Association between ipilimumab and celiac disease. Mayo Clin Proc.

[15] Scanlon SA, Murray JA (2011). Update on celiac disease—etiology, differential diagnosis, drug targets, and management advances. Clin Exp Gastroenterol.

[16] Lagana SM, Bhagat G (2019). Biopsy diagnosis of celiac disease. The Pathologist's Perspective in Light of Recent Advances Gastroenterol Clin North Am.

[17] Tarhini A (2013). Immune-mediated adverse events associated with ipilimumab CTLA-4 blockade therapy: the underlying mechanisms and clinical management. Scientifica..

[18] Geukes Foppen MH, Rozeman EA, van Wilpe S (2018). Immune checkpoint inhibition-related colitis: symptoms, endoscopic features, histology and response to management. ESMO Open.

[19] Wang Yinghong, Abu-Sbeih Hamzah, Mao Emily, Ali Noman, Qiao Wei, Trinh Van Anh, Zobniw Chrystia, Johnson Daniel Hartman, Samdani Rashmi, Lum Phillip, Shuttlesworth Gladis, Blechacz Boris, Bresalier Robert, Miller Ethan, Thirumurthi Selvi, Richards David, Raju Gottumukkala, Stroehlein John, Diab Adi (2018). Endoscopic and Histologic Features of Immune Checkpoint Inhibitor-Related Colitis. Inflammatory Bowel Diseases.

